# Monitoring of Antimicrobial Resistance to Aminoglycosides and Macrolides in *Campylobacter coli* and *Campylobacter jejuni* From Healthy Livestock in Spain (2002–2018)

**DOI:** 10.3389/fmicb.2021.689262

**Published:** 2021-07-02

**Authors:** Vicente Lopez-Chavarrias, Maria Ugarte-Ruiz, Carmen Barcena, Adolfo Olarra, Maria Garcia, Jose Luis Saez, Cristina de Frutos, Tania Serrano, Iratxe Perez, Miguel Angel Moreno, Lucas Dominguez, Julio Alvarez

**Affiliations:** ^1^VISAVET Health Surveillance Centre, Universidad Complutense de Madrid, Madrid, Spain; ^2^Facultad de Medicina Veterinaria y Zootecnia, Universidad Nacional Autónoma de México, Mexico City, Mexico; ^3^Subdirección General de Sanidad e Higiene Animal y Trazabilidad, Dirección General de la Producción Agraria, Ministerio de Agricultura, Pesca y Alimentación, Madrid, Spain; ^4^Laboratorio Central de Veterinaria (LCV Algete), Ministerio de Agricultura, Pesca y Alimentación, Madrid, Spain; ^5^TRAGSATEC, Tecnologías y Servicios Agrarios S.A., Madrid, Spain; ^6^Departamento de Sanidad Animal, Facultad de Veterinaria, Universidad Complutense de Madrid, Madrid, Spain

**Keywords:** *Campylobacter*, antibiotics, antimicrobial resistance, aminoglycosides, macrolides, flagellin, genes

## Abstract

Antimicrobial resistance (AMR) in *Campylobacter* spp. (*Campylobacter coli* and *Campylobacter jejuni*) is a concern due to its importance in public health, particularly when it involves aminoglycosides and macrolides, drugs of choice for treatment of human cases. Co-resistance to these two antimicrobial classes involves transfer of genetic elements and/or acquisition of mutations in different genetic loci, which can in turn spread through vertical or horizontal gene transfer (HGT) phenomena, with each route having different potential implications. This study aimed at evaluating the association between the presence of phenotypic resistance to these two antimicrobial classes in *C. coli* and *C. jejuni* recovered from livestock at slaughterhouses in Spain (as part of the AMR surveillance program), and at assessing the genetic heterogeneity between resistant and susceptible isolates by analysing the “short variable region” (SVR) of the *flaA* gene. Over the 2002–2018 period, antimicrobial susceptibility test results from 10,965 *Campylobacter* isolates retrieved from fecal samples of broilers, turkeys, pigs and cattle were collected to compare the proportion of resistant isolates and the Minimum Inhibitory Concentrations (MICs) against six antimicrobials including gentamicin (GEN), streptomycin (STR), and erythromycin (ERY). AMR-associated genes were determined for a group of 51 isolates subjected to whole genome sequencing, and the *flaA* SVR of a subset of 168 isolates from all hosts with different resistotypes was used to build a Neighbor-Joining-based phylogenetic tree and assess the existence of groups by means of “relative synonymous codon usage” (RSCU) analysis. The proportion of antimicrobial resistant isolates to both, aminoglycosides and macrolides, varied widely for *C. coli* (7–91%) and less for *C. jejuni* (all hosts 0–11%). Across hosts, these proportions were 7–56% in poultry, 12–82% in cattle, and 22–91% in pigs for *C. coli* and 0–8% in poultry and 1–11% in cattle for *C. jejuni*. Comparison of the MIC distributions revealed significant host-specific differences only for ERY in *C. jejuni* (*p* = 0.032). A significant association in the simultaneous presentation of AMR to both antimicrobial classes was observed across hosts/bacterial species. The *flaA* gene analysis showed clustering of isolates sharing resistotype and to a lesser degree bacterial species and host. Several resistance markers associated with resistance to aminoglycosides and macrolides were found among the sequenced isolates. The consistent association between the simultaneous presentation of AMR to aminoglycosides and macrolides in all hosts could be due to the persistence of strains and/or resistance mechanisms in *Campylobacter* populations in livestock over time. Further studies based on whole genome sequencing are needed to assess the epidemiological links between hosts and bacterial strains.

## Introduction

*Campylobacter coli* and *Campylobacter jejuni*, thermophilic bacteria of the genus *Campylobacter* spp., are the most frequently notified human gastrointestinal zoonotic pathogens in the European Union (EU) since 2005 (EFSA, 2005, 2006, 2007). Traditionally, *C. jejuni* was more frequently isolated from human cases than *C. coli*, but in 2017, 24.1% of *Campylobacter* confirmed human infections in the EU were caused by *C. coli* versus 22.2% due to *C. jejuni*, thus suggesting this pattern may vary (EFSA-ECDC, 2019b). Poultry and poultry products are considered the main source of human campylobacteriosis, followed by ruminants (beef, dairy cattle, and their manure) and environmental sources (Ravel et al., 2017; Rosner et al., 2017; An et al., 2018; Thepault et al., 2018). Consumption of pig meat has been linked to human cases caused by *C. coli* (Rosner et al., 2017). Although the prevalence of *Campylobacter* spp. in pork is high, previous studies found it was only associated with 2% of all human cases (Kittl et al., 2013), while beef has been linked to 19% of human cases (Boysen et al., 2014).

Treatment of human cases, when necessary, may be hampered by the increasing threat of antimicrobial resistance (AMR) observed in recent years (Friedrich, 2019). Macrolides [erythromycin (ERY) and azithromycin] and fluoroquinolones [ciprofloxacin (CIP)] are the drugs of choice in human patients requiring antibiotic treatment, but the latter class is not recommended for children. When these drugs are ineffective, systemic administration of aminoglycosides is the only option left (Bolinger and Kathariou, 2017; World Health Organization (WHO), 2017). Levels of AMR to aminoglycosides and macrolides in clinical thermophilic *Campylobacter* from humans are increasing (Aarestrup and Wegener, 1999; Bolinger and Kathariou, 2017; EFSA-ECDC, 2019b), and a similar trend for macrolides has been observed in isolates from pigs and broilers (Moore et al., 2006; Wang et al., 2016). In Europe, the proportion of resistant *Campylobacter* spp. isolates from food producing animals and humans vary depending on the country. Isolates originating from Spain showed higher levels of resistance to aminoglycosides [gentamicin (GEN) and streptomycin (STR)], macrolides (ERY), quinolones [CIP and nalidixic acid (NAL)], and tetracycline (TET) in *C. coli* from broilers, turkeys and pigs, and *C. jejuni* from broilers (EFSA, 2005, 2006, 2007; EFSA-ECDC, 2016, 2017, 2018a, 2019b). *C. coli* has traditionally shown higher levels of AMR to most antimicrobials compared with *C. jejuni* from both humans and animals (EFSA-ECDC, 2017, 2018a, 2019b). Therefore, the AMR problem may intensify if the importance of *C. coli* as a human pathogen keeps increasing. *C. coli* is now as prevalent as *C. jejuni* in broilers in some countries (Wieczorek and Osek, 2013), thus, monitoring of *C. coli* and *C. jejuni* AMR levels, particularly to macrolides and aminoglycosides, is equally important (EFSA-ECDC, 2018a, 2019b).

Resistance to aminoglycosides and macrolides can be mediated by multiple mechanisms, including chromosomal mutations and horizontal gene transferable elements (Davies and Wright, 1997; Saenz et al., 2000; Moore et al., 2006; Luangtongkum et al., 2009; Wieczorek and Osek, 2013; Bolinger and Kathariou, 2017). Antibiotic modifying enzymes (AMEs) are commonly involved in resistance to aminoglycosides (Saenz et al., 2000; Wieczorek and Osek, 2013; Garneau-Tsodikova and Labby, 2016), whereas ribosome methyltransferases (RMTs) are frequently related to resistance to macrolides (Saenz et al., 2000; Aarestrup, 2005). Co-resistance to both aminoglycosides and macrolides, as well as multidrug resistance (MDR) to additional antimicrobial classes, can be acquired through several mechanisms such as 16S rRNA RMTs (RmtB, ArmA) encoded in multi-drug resistance genomic islands (MDRGIs) carrying *erm* genes in *C. coli* (Aarestrup, 2005; Garneau-Tsodikova and Labby, 2016; Bolinger and Kathariou, 2017) and 23S rRNA RMTs (Saenz et al., 2000). Additional mechanisms can involve transferable genomic islands carrying multiple aminoglycoside resistance genes encoding AMEs in *C. coli* (Davies and Wright, 1997; Luangtongkum et al., 2009; Qin et al., 2012; Wieczorek and Osek, 2013) and multidrug macrolide efflux pumps (including the resistance enhancing *CmeABC* in *C. jejuni*), alone or in combination with target gene mutations (Aarestrup, 2005; Wieczorek and Osek, 2013; Garneau-Tsodikova and Labby, 2016; Bolinger and Kathariou, 2017). Although several of these mechanisms were first discovered in *C. coli*, evidence of transfer to *C. jejuni* was shown thereafter (EFSA-ECDC, 2018a).

Epidemiological studies complemented with genetic analyses are essential to help understand the mechanisms by which co-resistance and MDR to aminoglycosides and macrolides may be emerging in thermophilic *Campylobacter* from animals and humans (Luangtongkum et al., 2009; Wieczorek and Osek, 2013). Here, data from the national surveillance program on AMR in *Campylobacter* spp. from broilers, turkeys, pigs and cattle in Spain were analyzed to assess the prevalence of *Campylobacter* species in different animal hosts and the patterns of phenotypic AMR co-resistance to macrolides and aminoglycosides over the years. The results of this research can contribute to better explain AMR co-selection/MDR phenomena between these two antimicrobial classes in *Campylobacter* from livestock.

## Materials and Methods

### Study Population

The data analyzed here is based on sample collection, culture and antimicrobial susceptibility testing (AST) work carried out during 2002–2018 on isolates retrieved through the Spanish national veterinary AMR monitoring program for *Campylobacter* spp. in poultry (broilers and turkeys), pigs and cattle, according to EU legislation (EC, 2013). Samples for each animal species, originating from multiple farms, were collected at slaughterhouses covering 60% of the national throughput (Supplementary Figures 1, 2). Broiler samples were retrieved every year from 2002 to 2014 and every 2 years thereafter, turkey samples every 2 years from 2014 to 2018, pig samples every year from 2002 to 2013 and every 2 years thereafter, and cattle samples every year from 2007 to 2013 and every 2 years thereafter. Pooled samples collected every year for each host species ranged between 76 and 500 (mean = 228) for broilers, 467 and 500 (mean = 485) for turkeys, 171 and 384 (mean = 268) for pigs, and 163 and 384 (mean = 261) for cattle (Supplementary Table 1).

In relation to sampling and culture, pools made of samples from animals belonging to the same farm (from 10 caeca in poultry or from the caecum content of 2 animals in pigs and cattle) were collected at the slaughterhouse and transported refrigerated to the laboratory, where they were processed within 24 h after collection according to ISO 10272-2006-1. Single colonies with morphology compatible with *Campylobacter* spp. were identified as “*C. coli*,” “*C. jejuni*,” or “*C.* spp.” using API strips (up to 2010) and a multiplex PCR (2010 onward) (Ugarte-Ruiz et al., 2012).

Regarding AST, the AMR phenotype of *Campylobacter* spp. isolates was determined using the two-fold broth micro-dilution reference method (calculating Minimum Inhibitory Concentrations – MICs, according to ISO Norm 20776-1:2006) or diffusion technique (calculating Inhibition Zone Diameters – IZDs) (Ugarte-Ruiz et al., 2015).

Antimicrobial susceptibility testing results for the six antimicrobials listed on the AMR surveillance programs for *Campylobacter* spp. in the EU (EC, 2013) were available for isolates from all species and years: CIP, TET, NAL, STR, ERY, and GEN. For ERY in broilers, IZDs were used up to 2004 (included) and MICs were used thereafter. For STR in broilers and pigs, IZDs were used up to 2005 (included) and MICs were used thereafter.

Samples in which information on culture result, molecular identification and/or AMR typing was missing were excluded from the analysis. The following information was available for all samples in the study: host species, *Campylobacter* growth result, *Campylobacter* species, year and AST result. Isolates were classified as “susceptible” (wild-type strains) or “not susceptible” (resistant strains) according to epidemiological cut-off points (ECOFFs) provided by the “European Committee on Antimicrobial Susceptibility Testing” (EUCAST)^1^ (Table 1). Proportions of resistant isolates for each bacterial and host species and period were defined as very low (<1%), low (1.1–10%), moderate (10.1–20%), high (20.1–50%), very high (50.1–70%), and extremely high (70.1–100%), as recommended by EFSA (EFSA-ECDC, 2019b).

**TABLE 1:** Epidemiological cut-offs (ECOFFs) used for interpretation of MICs in *Campylobacter* spp. (Source EUCAST).

**Antimicrobial**	***C. coli* MIC (>) Micro-dilution**	***C. coli* DIAM (<) Difusion**	***C. jejuni* MIC (>) Micro-dilution**	***C. jejuni* DIAM (<) Difusion**
Gentamicin (GEN)	2	NA	2	20
Streptomycin (STR)	4	13	4	13
Erythromycin (ERY)	8	24	4	22
Ciprofloxacin (CIP)	0,5	26	0,5	26
Nalidixic acid (NAL)	16	NA	16	NA
Tetracycline (TET)	2	30	1	30

### Statistical Analyses

Proportions of resistant *C. coli* and *C. jejuni* isolates to each antimicrobial from the different hosts were compared using Z-tests, adjusted for multiple comparisons by the Holm-method. Cochrane-Armitage logistic regressions were used to test for trends of AMR phenotypic resistance in *C. coli* and *C. jejuni* per antimicrobial and host species, and the relative change in the proportion of resistant isolates per year was computed along with its 95% confidence interval. The association in the simultaneous presentation of phenotypic resistance to STR/ERY, GEN/ERY, and GEN/STR over the whole study period and in different time periods (2002–2006, 2007–2012, and 2013–2018) was further evaluated for each bacterial and host species using relative risks and chi-squared and Fisher’s exact tests.

In order to evaluate differences in the distribution of MICs values in *C. coli* and *C. jejuni* from the four host species, available data were represented as “squashtograms.” The existence of statistical differences in MICs distributions in susceptible and not-susceptible (here referred to as “resistant”) isolates depending on bacterial species (for a given host) or on host (for a given bacterial species) was evaluated using Mann–Whitney *U* or Kruskal–Wallis tests followed by Dunn’s *post hoc* tests, correcting for multiple comparisons by the Benjamini-Hochberg method.

### Molecular Characterization Based on *flaA* Sequencing

A subset of 125 isolates including all combinations of hosts, bacterial species, year of recovery and AMR phenotype, randomly chosen within each category, was used to assess their genetic relatedness by comparing the flagellin *flaA* short variable region (SVR) gene sequence as described by other authors (Ugarte-Ruiz et al., 2013; Zhang et al., 2018). Selected isolates were classified into two categories: isolates with simultaneous phenotypic resistance to aminoglycosides and ERY (*n* = 53, “cases”) and isolates not presenting this simultaneous resistance (*n* = 72, “controls”). Amplification of the *flaA* SVR gene sequence by PCR was performed as previously described (Ugarte-Ruiz et al., 2013), and the obtained amplicons were sequenced. Additionally, the *flaA* sequence of fifty-one isolates (8 “cases” and 43 “controls”) previously subjected to whole genome sequencing (GenBank accession codes SRX5575129 to SRX5587545) was extracted (along with information on the presence of resistance genes) using a homemade Python script. The resulting 176 sequences were then aligned using MUSCLE (Edgar, 2004) and a Neighbour-Joining (NJ) phylogenetic tree with 1,000 bootstraps was built to evaluate the phylogenetic relationship between isolates. The *flaA* gene of the NCTC 1168 *C. jejuni* strain (1719 nucleotides-long, bacterial chromosome positions 1269232 to 1270950) was used as an external reference. A multiple correspondence analysis (MCA) of the relative synonymous codon usage (RSCU) values categorized as > 1 (positive bias) or < 1 (negative bias) was performed as described before (Meinersmann et al., 2005). The MCA included, along with the RSCU of variable codons, other available covariates (bacterial species, host species, resistance to GEN, ERY, and STR, and clade as determined in the NJ phylogenetic tree).

**FIGURE 1:**
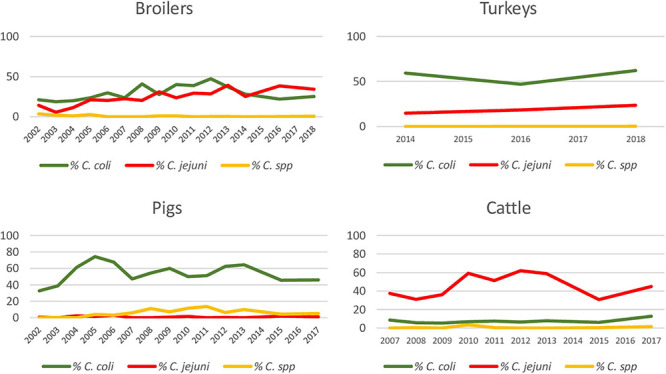
Percentage of *Campylobacter* isolates over total sampled, identified as *C. coli*, *C. jejuni*, and *C.* spp. for each host species throughout study period.

Microsoft Access was used for data handling and database initial analyses. Data were further handled with Microsoft Excel and imported into “R” version 3.6.3 (R Core Team, 2020). The R packages “FSA” (Ogle et al., 2020), “plyr” (Wickham, 2011), and “ggplot2,” “dplyr,” “reshape2,” and “tidyr” (Wickham et al., 2019) were used for the analysis and visual representation of the data. Information on resistance-associated markers from the sequenced strains was extracted using ResFinder (Bortolaia et al., 2020). MEGA-X (Kumar et al., 2018) and DnaSP6 (Rozas et al., 2017) were used on imported DNA sequences for the preparation and analysis of sequence alignments. R packages “BiocManager” (“coRdon”) (Morgan and Ramos, 2019) and “seqinr” (Charif and Lobry, 2007) were used for the calculation of RSCUs. “Corrplot” (Wei et al., 2017), “FactoMineR” (Lê et al., 2008), “factoextra” (Kassambara and Mundt, 2020), and “ggtheme” were used for the MCA analysis. All Figures were generated using R except Figure 1 (Excel) and Figure 6 (MEGA-X).

## Results

In total 3,413 independent samples from broilers, 1,455 from turkeys, 3,750 from pigs and 2,347 from cattle were included in the analysis, of which 2,000 (58.6%), 1,090 (74.9%), 2,218 (59.2%), and 1,273 (54.3%) resulted in the isolation of *Campylobacter* spp., respectively (Table 2). The number of samples analyzed, isolates recovered, and isolates subjected to AST varied depending on year and host species (Supplementary Table 2).

**TABLE 2:** Collection period, number of fecal samples and positive samples for *Campylobacter* isolation from each host species included in the study.

**Host species**	**Broilers**	**Turkeys**	**Pigs**	**Cattle**
Years	2002–2018	2014–2018	2002–2017	2007–2017
Sample size	3,413	1,455	3,750	2,347
*C. coli*	1,023 (30.0%)	815 (56.0%)	1,968 (52.5%)	183 (7.8%)
*C. jejuni*	957 (28.0%)	273 (18.8%)	33 (0.9%)	1,074 (45.8%)
*C.* spp.	20 (0.6%)	2 (0.1%)	217 (5.8%)	16 (0.7%)

Over the entire study period, *C. coli* was the most frequently isolated species in pig (88.7%; 1,968/2,218) and turkey (74.8%; 815/1,090) samples, while *C. jejuni* was the most frequent species in cattle (84.4%; 1,074/1,273). In broilers, the proportion of *C. coli* and *C. jejuni* was very similar (51.2%; 1,023/2,000 and 47.8%; 957/2,000, respectively) (Table 2).

Although there were differences depending on the year, the proportion of positive samples to *C. coli* and *C. jejuni* remained relatively constant over the whole study period in pigs and cattle, with one bacterial species being more prevalent than the other one, while the situation was more variable in broilers (Figure 1). Significant increasing trends were observed in the proportion of positive samples for *C. jejuni* in broilers and turkeys, with annual-biannual rates of increase of 9.7% (95%CI: 6.16–13.33%) and 26.2% (95%CI: 15.81–37.43%), respectively (Figure 1).

**FIGURE 2:**
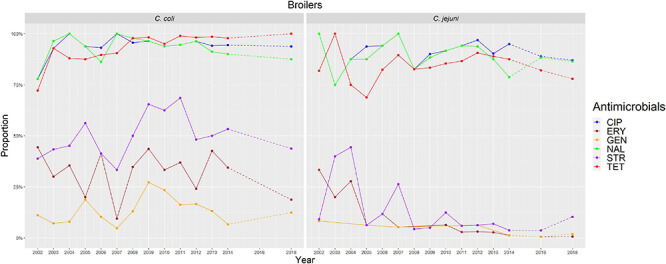
Graphical representation of AMR proportions to each antimicrobial in *C. coli* and *C. jejuni* from broilers for 2002–2018. Years in which AST was performed are indicated in the X axis; dashed lines indicate periods in which AST was not performed every year (no AST performed in *C. coli* in 2016).

**FIGURE 3:**
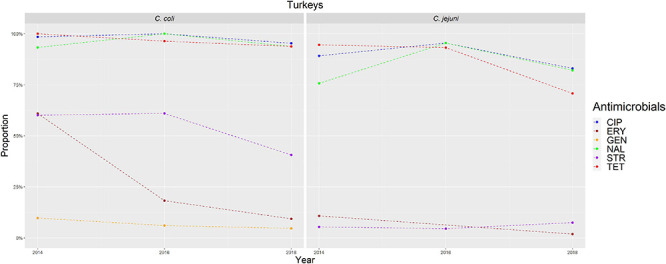
Graphical representation of AMR proportions to each antimicrobial in *C. coli* and *C. jejuni* from turkeys for 2014–2018. Years in which AST was performed are indicated in the *X*-axis; dashed lines indicate periods in which AST was not performed every year.

**FIGURE 4:**
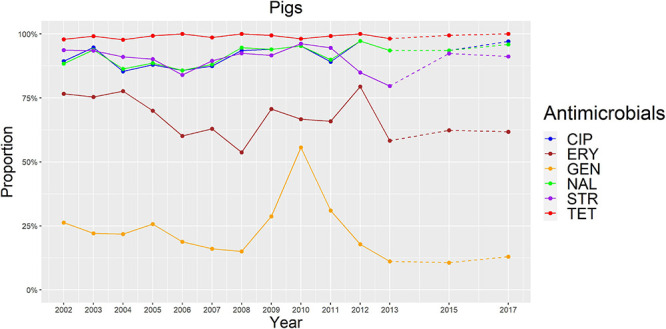
Graphical representation of AMR proportions to each antimicrobial in *C. coli* from pigs for 2002–2017. Years in which AST was performed are indicated in the *X*-axis; dashed lines indicate periods in which AST was not performed every year.

**FIGURE 5:**
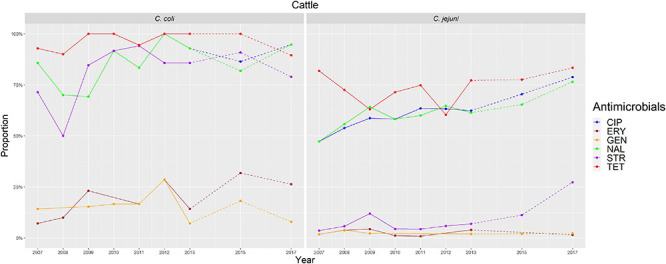
Graphical representation of AMR proportions to each antimicrobial in *C. coli* and *C. jejuni* from cattle for 2007–2017. Years in which AST was performed are indicated in the *X*-axis; dashed lines indicate periods in which AST was not performed every year.

The overall proportion of isolates resistant to CIP, NAL, and TET was extremely high (>80%) in both *C. coli* and *C. jejuni* from all host species (Table 3 and Figures 2–5), with yearly values exceeding 70% throughout the study period except in *C. jejuni* from cattle (Figure 5). Still, there were significant differences in the proportion of resistant isolates to these three antimicrobials depending on the host (Table 3). The proportion of CIP and NAL-resistant *C. coli* isolates was significantly lower in cattle compared with broilers and turkeys (*p* < 0.05) (and in pigs compared with turkeys for CIP, *p* < 0.001). In the case of TET, *C. coli* isolates from pigs were significantly more resistant than *C. coli* from cattle and broilers (*p* < 0.001), although resistance was still above 95% in all species (Table 3). In the case of *C. jejuni*, cattle isolates were significantly less resistant to the three antimicrobials compared with isolates from poultry (*p* < 0.05).

**TABLE 3:** Percentage of *Campylobacter* isolates not susceptible (resistant) to each antimicrobial in the four host species throughout the studied period.

**Antimicrobials**		**Tetracycline**	**Nalidixic acid**	**Ciprofloxacin**	Streptomycin*	Erythromycin*	**Gentamicin**
		*Coli* (MIC > 2)	*Coli*/*Jejuni* (MIC > 16)	*Coli*/*Jejuni* (MIC > 0.5)	*Coli*/*Jejuni* (MIC > 4; DIAM < 13)	*Coli* (MIC > 8; DIAM < 24)	*Coli*/*Jejuni* (MIC > 2)
		*Jejuni* (MIC > 1)				*Jejuni* (MIC > 4; DIAM < 22)	
*C. coli* (%)	Broilers (*n* = 634)	95.9^a^	93.3^a^	94.5^a,b^	54.7^a^	34.8^a^	14.7^a^
	Turkeys (*n* = 279)	97.5^a,b^	95.3^a^	98.2^b^	55.9^a^	36.6^a^	7.5^b^
	Pigs (*n* = 1,692)	99.1^b^	91.7^a,b^	91.7^a,c^	90.6^b^	66.6^b^	22.2^c^
	Cattle (*n* = 149)	95.3^a^	86.7^b^	87.3^c^	82.0^c^	19.3^c^	12.0^a,b^
*C. jejuni* (%)	Broilers (*n* = 772)	83.1^a^	88.5^a^	91.1^a^	7.7^a^	2.9^a^	1.0^a^
	Turkeys (*n* = 231)	83.1^a^	86.1^a^	88.7^a^	6.1^a^	2.6^a^	0.0^a^
	Cattle (*n* = 828)	74.1^b^	63.1^b^	63.8^b^	10.2^a^	1.7^a^	1.4^a^

Different superscripts indicate significant differences between hosts for each bacterial species

*Diffusion technique: streptomycin in broilers and pigs (2002–2005) and erythromycin in broilers (2002–2004).

The proportion of resistance to the remaining three antimicrobials (STR, ERY, and GEN) was much more variable (Table 3 and Figures 2–5). In the case of STR, extremely high (80–91%) or high to very high (∼55%) levels were found in *C. coli* from cattle and pigs and from broilers and turkeys, respectively, with significant differences between all hosts species except between broilers and turkeys (pigs > cattle > poultry). In contrast, values <11% were observed in *C. jejuni* from all three host species from which this bacterial species was recovered (broilers, turkeys, and cattle). Although levels of resistance in *C. jejuni* were always significantly lower than in *C. coli* for any given host species, no significant differences between host species were observed.

The proportion of resistance to ERY in *C. coli* was very high (67%) for pigs, high (35%) for broilers and turkeys, and moderate (19%) for cattle (pigs > poultry > cattle) (Table 3 and Figures 2–5). Overall values in *C. jejuni* from all host species were < 3% and significantly lower than those from *C. coli*, again with no significant differences across hosts.

Finally, the proportion of GEN resistant-isolates was low (<25%) in *C. coli* from all species (Table 3 and Figures 2–5), although the proportion in pigs was again significantly higher than that observed in other host species (*p* < 0.001). Resistance levels in *C. jejuni* were lower (<2%) and significantly different from those observed in *C. coli* from the same host and, once more, no significant differences between hosts were observed.

Significant (*p* < 0.05) trends in the proportion of cattle resistant isolates were observed associated to increasing annual rates for ERY and *C. coli* (29.9% although with a wide 95%CI: −31.36 to 145.77) and STR and *C. jejuni* (16.7%, 95%CI: 2.89–32.28) (Figure 5). Significant (*p* < 0.001) trends in the proportion of resistant *C. jejuni* isolates recovered yearly from broilers were also observed for two antimicrobials, in both cases associated to decreasing annual rates: STR (−9.9%, 95%CI: −16.13 to −3.14) and ERY (−27.5%, 95%CI: −44.60 to −5.10) (Figure 2).

Other significant (*p* < 0.001) trends were found associated with modest annual rates of increase in *C. jejuni* in cattle for CIP (5.22%, 95%CI: 3.91–6.54) and NAL (4.11%, 95%CI: 2.20–6.06), in *C. coli* in broilers for TET (1.61%, 95%CI: 0.93–2.29), and in *C. coli* in pigs for CIP (0.64%, 95%CI: 0.20–1.09). For the rest of antimicrobials, host and bacterial species no significant trends were detected (Figures 2–5).

For any given bacterial species, an analysis of the quantitative AST results across hosts species revealed significant differences (*p* = 0.032) in the distribution of MIC values between “susceptible” and “not susceptible” isolates only for *C. jejuni* strains from turkeys “not susceptible” to ERY (MIC ≥ 256 mg/L) (Supplementary Excel File 1). In contrast, for any given host species, no significant differences were observed between MIC distributions of “susceptible” and “not susceptible” *C. coli* vs. *C. jejuni* isolates.

### Co-resistance and MDR Phenotypic Profiles

The main resistance profiles observed in each bacterial and host species are shown on Supplementary Table 3. Of all *C. coli* isolates from all host species, >85% were resistant to three (CIP-TET-NAL, TET-ERY-STR) or more antimicrobials and >60% were resistant to three or more antimicrobial classes (MDR). The most common resistance profiles for *C. coli* from each host were CIP-TET-NAL and CIP-TET-NAL-STR in broilers and turkeys (18–28% of all isolates in each host species for each profile), CIP-TET-NAL-STR in cattle (∼50% of all *C. coli* isolates) and CIP-TET-NAL-STR-ERY in pigs (∼40% of all *C. coli* isolates). The proportion of pan-susceptible isolates for *C. coli* in all host species was low (0–4%), and lower than the proportion of resistant isolates to all six antimicrobials (0–16%).

In comparison, the proportion of *C. jejuni* isolates from all hosts resistant to three or more antimicrobials was 54–76% whereas the MDR proportion was 6–9%. The most common resistance profile for *C. jejuni* from all hosts was CIP-TET-NAL, amounting to between ∼45% of all cattle and 60–70% of all broiler and turkey isolates. In this case, the proportion of pan-susceptible isolates (5–15%) was higher than that of resistant isolates to the six antimicrobials in all species (<1%).

### Association Between Resistance to GEN, STR, and ERY

Overall, a significant association between the occurrence of phenotypic resistance to aminoglycosides and macrolides was observed, so that *C. coli* and *C. jejuni* from all host species (except *C. coli* in pigs and turkeys and *C. jejuni* in turkeys) resistant to one of the two aminoglycosides (or to both) were more likely to be also resistant to ERY (Table 4). This association was stronger in *C. jejuni* and/or cattle isolates.

**TABLE 4:** Association between phenotypic resistance to gentamicin, streptomycin, and erythromycin in *C. coli* and *C. jejuni* isolates from livestock.

**Host**	**Bacterial species**	***N***	**Erythromycin-R (%)**	**Antimicrobial**	**Resistance (%)**	**Resistance among EryR (%)**	***p*-value**	RR*
Broilers	*C. coli*	634	34.5	Streptomycin	54.4	68.9	<0.001	1.86
				Gentamicin	14.7	24.7	<0.001	1.90
	*C. jejuni*	772	2.5	Streptomycin	7.6	31.6	<0.001	5.58
				Gentamicin	1.0	15.8	<0.001	17.90
Pigs	*C. coli*	1692	66.7	Streptomycin	90.7	90.7	0.953	1.00
				Gentamicin	22.0	25.0	<0.001	1.18
Turkeys	*C. coli*	279	36.6	Streptomycin	56.0	66.7	0.008	1.58
				Gentamicin	7.5	10.8	0.156	1.48
	*C. jejuni*	231	2.6	Streptomycin	6.0	33.3	0.045	7.75
				Gentamicin	0.0	0.0	1	17.85
Cattle	*C. coli*	149	19.5	Streptomycin	82.6	96.6	0.028	5.92
				Gentamicin	12.1	31.0	0.002	3.27
	*C. jejuni*	828	1.7	Streptomycin	10.3	78.6	<0.001	32.05
				Gentamicin	1.4	28.6	<0.001	27.20

*Relative risk of presenting resistance to erythromycin in isolates resistant to streptomycin/gentamicin.

### Analysis of *flaA* and AMR Genes

**FIGURE 6:**
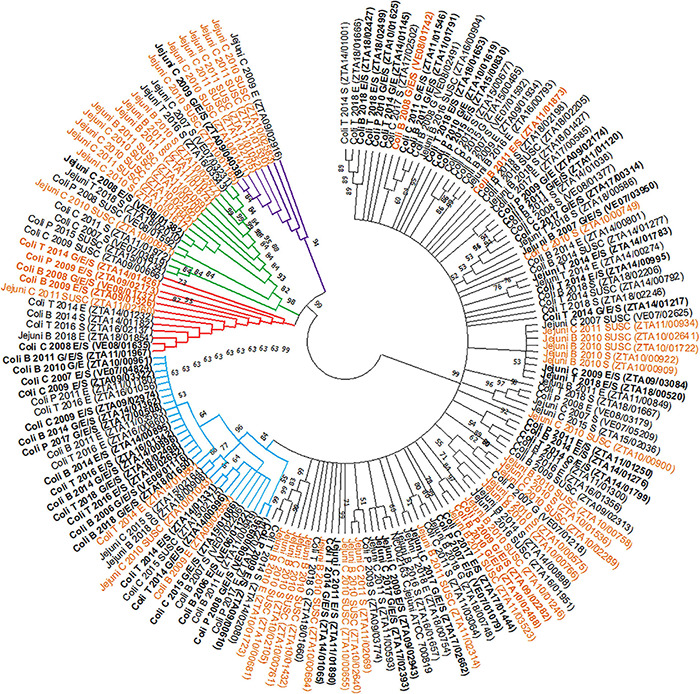
Phylogenetic tree of the short variable region (SVR) of *flaA* genes from 168 selected *Campylobacter* isolates, combining bacterial species, host, year of recovery and AMR phenotype, obtained by the Neighbour-Joining method [Group 1 = black; Group 2 = blue; Group 3 = red; Group 4 = green; Group 5 = violet; NC002163 = Reference strain; B = Broilers; T = Turkeys; P = Pigs; C = Cattle; G = Gentamicin; E = Erythromycin; S = Streptomycin; SUSC = susceptible isolates; aminoglycosides/macrolides co-resistant strains (“cases”) in bold; strains subjected to WGS in orange].

Over 300 bp (including the 267 bp-long SVR used in the analysis) of the *flaA* gene sequence were correctly determined in 168 of the 176 chosen isolates (all except 2 “cases” and 6 “controls”). Figure 6 displays the phylogenetic tree constructed using the selected final 168 isolates (59 “cases” and 109 “controls”) plus the reference strain. Overall, a total of 127 single nucleotide polymorphisms (SNPs) located in 100 polymorphic sites were found, leading to 73 unique *flaA* SVR gene sequences. The haplotype diversity (Hd – probability that two randomly selected sequences are different) was 0.975, and every two sequences differed on average by 27 SNPs with an overall mean evolutionary distance (d) between the two sequences of 0.09.

The 168 isolates were classified into five groups based on the topology of the tree: group 1, including the majority of the sequenced isolates (*n* = 100 isolates), group 2 (*n* = 31 isolates), group 3 (*n* = 12 isolates), group 4 (*n* = 17 isolates), and group 5 (*n* = 8 isolates) (Figure 6). Groups 2–5 formed separate clades from group 1 (bootstrap > 60). Groups 2 and 3, consisting mainly of *C. coli* strains (28/31 and 10/12, respectively) predominantly from poultry (>60%), showed similar proportions of isolates resistant to aminoglycosides and macrolides (“cases”) and of “controls” (18/31 and 6/12, respectively) (Table 5). Groups 4 and 5 showed a higher proportion of *C. jejuni* isolates (13/17 in group 4 and 8/8 in group 5) from cattle (9/17 and 8/8), and the frequency of isolates with simultaneous resistance to both antimicrobial classes (“cases”) in these groups was much lower (1/17 and 1/8, respectively) (Table 5).

**TABLE 5:** Numbers and proportions of isolates included in each of the groups formed from the phylogenetic analysis based on phenotypical AMR susceptibility, bacterial species, host species, and case/control categories.

**Group #**	**1**	**2**	**3**	**4**	**5**	**Total**
*N*	(*n* = 100)	(*n* = 31)	(*n* = 12)	(*n* = 17)	(*n* = 8)	(*n* = 168)
GEN-R	22 (22.0%)	**11 (35.5%)**	**3 (25.0%)**	0 (0.0%)	1 (12.5%)	37 (22.0%)
ERY-R	45 (45.0%)	**26 (83.9%)**	**8 (66.7%)**	1 (5.9%)	2 (25.0%)	82 (48.8%)
STR-R	58 (58.0%)	**19 (61.3%)**	**8 (66.7%)**	10 (58.8%)	1 (12.5%)	96 (57.1%)
*Coli*	54 (54.0%)	**28 (90.3%)**	**10 (83.3%)**	4 (23.5%)	0 (0.0%)	96 (57.1%)
*Jejuni*	46 (46.0%)	3 (9.7%)	2 (16.7%)	**13 (76.5%)**	**8 (100.0%)**	72 (42.9%)
Broilers	37 (37.0%)	**12 (38.7%)**	**5 (41.7%)**	4 (23.5%)	0 (0.0%)	58 (34.6%)
Cattle	27 (27.0%)	7 (22.6%)	3 (25.0%)	**9 (52.9%)**	**8 (100.0%)**	54 (32.1%)
Pigs	**14 (14.0%)**	**4 (12.9%)**	1 (8.3%)	2 (11.8%)	0 (0.0%)	21 (12.5%)
Turkeys	22 (22.0%)	**8 (25.8%)**	**3 (25.0%)**	2 (11.8%)	0 (0.0%)	35 (20.8%)
Cases	33 (33.0%)	**18 (58.1%)**	**6 (50.0%)**	1 (5.9%)	1 (12.5%)	59 (35.1%)
Controls	67 (67.0%)	13 (41.9%)	6 (50.0%)	**16 (94.1%)**	**7 (87.5%)**	109 (64.9%)

*In bold = predominant proportions within each group and category.*

**FIGURE 7:**
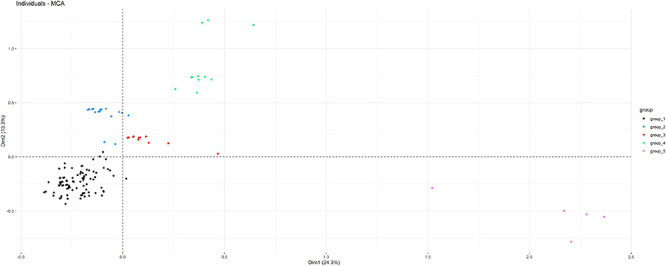
Distribution of the 168 isolates subjected to *flaA* gen sequencing according to the first two dimensions of a multiple correspondence analysis (MCA) performed considering information on the RSCU of variable codons, bacterial and host species, resistance to gentamicin, erythromycin, and streptomycin, and clade as determined in the NJ phylogenetic tree (observations are colored according to the clade).

Only complete RSCU values from the 20 variable codons among the 168 isolates were included in the MCA analysis. The first two dimensions identified in the MCA explained 38% of the total variability observed. Isolates included in each of the five groups identified in the phylogenetic tree were also clustered according to the first two dimensions of the MCA (Figure 7).

Different resistance markers involved in AMR against macrolides and aminoglycosides were found in the isolates subjected to WGS. Several genes involved in the CmeABC efflux pump (*cmeB*, *cmeC*, and *cmeR*) were present in 39–40/40 sequenced *C. jejuni* isolates and missing in 10–11/11 *C. coli* strains, while the *cmeA* gene was present in 34/40 and 4/11 of the *C. jejuni* and *C. coli* strains, respectively, but their presence was not associated with ERY resistance (Supplementary Excel File 2). Among the 12 ERY-resistant isolates, three presented mutations associated with macrolide-resistance in the 23S rRNA encoding gene and other three carried the *erm*(B) gene, while no resistance marker was found in the remaining six isolates. Regarding aminoglycoside-resistance associated genes, seven different genes were found in one or more strains (between 0 and 3 per strain), and their presence was associated with resistance to STR and/or GEN except in one susceptible *C. jejuni* strain from cattle (Supplementary Excel File 2). No apparent association between a specific *flaA* gene group and the presence of any of the resistance markers was observed.

## Discussion

Antimicrobial resistance is becoming a major problem for the treatment of diseases caused by zoonotic bacteria such as thermophilic *Campylobacter*. The mechanisms by which AMR can spread in a bacterial population (vertically or horizontally) has enormous implications, since it can determine the speed at which AMR phenotypes disseminate. Of particular concern are genetic traits conferring MDR (Magiorakos et al., 2012), particularly when transmitted together. Hence, it is of paramount importance to explore the genetic mechanisms implicated in AMR in *C. coli* and *C. jejuni* from the different hosts involved in the epidemiology of infection in humans (EFSA-ECDC, 2020). As described elsewhere, this study of isolate-based phenotypic data versus aggregated data has also proven to be a reliable means of gaining insight into such mechanisms (Alvarez et al., 2020), and the assessment of phenotypic susceptibility patterns found here can guide the genetic analysis in a “top-down” approach (Sheppard and Maiden, 2015).

The annual proportion of *Campylobacter* positive samples found in our samples from broilers collected over a 17-year period (ranging from 26.2 to 76.7%) was higher than values reported by EFSA from EU member states (26%) (EFSA-ECDC, 2019a) and mostly higher than values reported in other regions of the world such as China (30.2%) (Tang et al., 2020). The proportion of *Campylobacter* positive samples in cattle from 2007 to 2017 (37–69.5%) was also much higher than values reported by EFSA for 10 EU countries (1.5–3.5%) (EFSA-ECDC, 2018b, 2019a), although country-specific studies in Finland (Hakkinen et al., 2007), and Lithuania (Ramonaite et al., 2013) reported values more similar to the ones found here (39.6 and 80%, respectively). Similarly, the percentage of pig samples from which *Campylobacter* isolates were retrieved in our study (33.4–80%) was in the range of results reported for Greece (49.1%) (Papadopoulos et al., 2020), much higher than previously reported by EFSA for 8 EU countries (2–7%) (EFSA-ECDC, 2018b, 2019a) and lower than reported in a Danish study (92%) (Boes et al., 2005). However, the percentages we found in turkeys (65.4–85.9%) were similar to an EU report comprising 5 countries (71.6%) (EFSA-ECDC, 2019a) but lower than found in a German study (90–100%) (Ahmed et al., 2016).

As expected, the host species were strongly associated with the *Campylobacter* species retrieved in positive samples, although proportions found for each bacterial species may vary depending on isolation protocols used. The predominance of *C. coli* in pig samples found in our study is in agreement with previous studies from Denmark (Boes et al., 2005). However, and even though this bacterial species has been traditionally associated with pigs, it is becoming more common in poultry (Miller et al., 2006). In our collection, *C. coli* was in fact the predominant species in turkey, while a more balanced distribution between *C. coli* and *C. jejuni* was reported in turkey samples from Germany (Ahmed et al., 2016). In broilers, a close to 50/50 distribution for *C. coli*/*C. jejuni*, as the one found here, was also observed in samples from China (Tang et al., 2020). However, EFSA reported a predominance of *C. jejuni* with 2,452 *Campylobacter* positive samples from 16 countries (EFSA-ECDC, 2019a). In cattle, a study from Denmark (Nielsen et al., 1997) found similar proportions for each bacterial species (6.8% *C. coli*, 90.9% *C. jejuni*, and 2.3% *C.* spp.) than our study (14.4% *C. coli*, 84.4% *C. jejuni*, and 1.2% *C.* spp.).

As presumed, the level of resistance to the antimicrobials used in our study was closely linked with the *Campylobacter* bacterial species found, with higher levels of resistance in *C. coli* than in *C. jejuni* in agreement with previous research (Pergola et al., 2017; Alvarez et al., 2020). The lack of barriers to horizontal gene transfer (HGT) in *C. coli* may explain the higher levels of MDR observed in this bacterial species compared to *C. jejuni* (Pearson et al., 2015).

Out of the six antimicrobials assessed here, high to extremely high levels of resistance were found for three of them (CIP, NAL, and TET) in *C. coli*, while in *C. jejuni* they ranged between medium to very high. For CIP and NAL these levels were consistently higher than those described for isolates from food animals in other European countries with the exception of *C. jejuni* in turkeys (equal levels to Italy, Poland and Portugal at 70%) and cattle (equal levels to Italy at 80%) (EFSA-ECDC, 2020). For TET in *C. jejuni* in cattle, levels in Spain (85%) were between levels reported in Austria, Denmark and the Netherlands (60%) and levels reported in Italy (95%) (EFSA-ECDC, 2020).

Resistance levels to the remaining three antimicrobials analyzed in this study were much more variable, yet again consistently higher than in other European countries across hosts and bacterial species. The exceptions were *C. jejuni* from turkeys (15% ERY in Portugal vs. 2.6% in Spain; 20% STR in Poland vs. 6.1% in Spain) and cattle (10% ERY in Italy vs. 1.7% in Spain) (EFSA-ECDC, 2020).

Overall, a significant association between the presentation of phenotypic resistance to ERY (macrolide) and STR and GEN (aminoglycosides) was consistently found for both *C. coli* and *C. jejuni* from most host species (Table 3). When the association between antimicrobial pairs was analyzed stratifying by time-periods (2002–2006, 2007–2012, and 2013–2018) certain categories were not significantly associated, probably due to being smaller sample sizes (data not shown). Unsurprisingly, STR-resistant isolates had a significantly higher probability of being also resistant to GEN, which was expected given that they belong to the same antimicrobial class (aminoglycosides) and therefore share resistance mechanisms, mostly based on natural transformation, homologous recombination and sharing of MGEs (Davies and Wright, 1997; Luangtongkum et al., 2009; Qin et al., 2012; Wieczorek and Osek, 2013).

*Campylobacter* is considered a high-risk pathogen in terms of AMR due to the high levels of HGT and the association of AMR genes in MDRGIs. Some authors argue that the transfer of MDRGIs is likely to lead to co-selection phenomena after their genetic mobilization. This could explain why *Campylobacter* adapts so quickly in its interaction with the host, constantly obtaining improved phenotypes (Sheppard and Maiden, 2015).

The *erm*(B) gene, previously described only in Asia (Qin et al., 2014) and possibly originating from Gram-positive bacteria, was found in Spain in one *C. coli* from broiler in 2015 (Florez-Cuadrado et al., 2016) and two *C. coli* from turkeys in 2017 (Florez-Cuadrado et al., 2017). This was the first European report of this gene, associated with other genes in MDRGIs bearing resistance to ERY, CIP, TET, and NAL, and involved in AMR to STR (and present in isolates that may be susceptible to GEN). The three *erm*(B)-carrying strains, included in this study, were found in isolates showing simultaneous resistance to aminoglycosides and were clustered in different clades (1, 2, and 3). However, given the very limited number of ERY-resistant sequenced strains no conclusions can be drawn regarding their association with specific genetic populations. The inclusion of *erm*(B) genes in plasmids encoding additional resistance genes to other antibiotics in *C. coli* from food animals could pave the way to rapid dissemination of macrolide resistance (EFSA-ECDC, 2018a, 2019b). Besides, reported resistance levels to ERY in humans have been consistently higher for *C. coli* than for *C. jejuni* (EFSA-ECDC, 2018a), and similar reports have been made in poultry (Pergola et al., 2017) in agreement with our findings. Since macrolides are one of the three “Critically Important Antimicrobial” classes used for the treatment of human campylobacteriosis (along with fluoroquinolones and aminoglycosides) (World Health Organization (WHO), 2017), a more in-depth knowledge into their resistance mechanisms is warranted.

The increasing rates of resistance to ERY in *C. coli* and to STR in *C. jejuni* of cattle origin described here suggest this host species could play an increasingly important role in the epidemiology of AMR in *Campylobacter*. A nationwide case-control study carried out in Luxembourg identified beef consumption as an important source of infection for *C. coli* (Mossong et al., 2016), thus suggesting that cattle may be a relevant reservoir for this foodborne pathogen.

MICs values in isolates classified as “susceptible” or “not susceptible” may indicate the presence or absence of different AMR determinants in the bacterial genome. The significantly higher MICs values observed in this study for ERY in turkey resistant isolates (Supplementary Excel File 1) could indicate the presence of the transferable *erm*(B) gene. However, out of the 12 ERY-resistant isolates subjected to WGS, only three carried the *erm*(B) gene (and had MICs ranging between 32 and 256 ug/ml), and additionally, mutations in the 23S rRNA encoding gene were found in just three isolates (Supplementary Excel File 2). This suggests that other mechanisms may be involved in the observed increased MICs in certain isolates, such as mutational resistance affecting the expression of the CmeABC efflux pump in *C. jejuni* (Zhang et al., 2017). This, linked with the high proportion of ERY-resistant isolates found in *C. coli* from turkeys in other European countries (EFSA-ECDC, 2017, 2020) further highlights the need of clarifying the resistance mechanisms present in resistant isolates from this host. In fact, EFSA recommends investigating the molecular mechanisms of macrolide resistance, especially in isolates resistant to high concentrations of ERY, in order to detect chromosomal mutations or the presence of the transferable *erm*(B) gene (EFSA-ECDC, 2019b). Furthermore, these same guidelines recommend searching for ERY resistant genes, not only in resistant strains presenting concomitant resistance to aminoglycosides or a MDR phenotype, but also in susceptible isolates. Thus, an in-depth characterization of resistant isolates would be needed to confirm this hypothesis. The integration of phenotypic and genomic analyses may allow predicting differences in resistance levels beyond resistance thresholds (Bolinger and Kathariou, 2017; EFSA-ECDC, 2019b).

Phylogenetic studies based on *flaA* SVR gene sequencing have been used in the past to study the epidemiology of *Campylobacter* spp. from different sources (Zhang et al., 2018). Previously, studies based on the *flaA* gene sequence had not found a relationship between AMR and specific genotypes (Corcoran et al., 2006). However, in our strain collection five distinct groups were identified, two of which were associated with an increased proportion of simultaneous resistance to aminoglycosides and macrolides (groups 2 and 3), predominantly formed by *C. coli* isolates from broilers and turkeys (Table 5). In contrast, isolates in groups 4 and 5 were primarily *C. jejuni* of cattle origin. The existence of “cattle specialist *C. jejuni* lineages” has been previously speculated, implying that adaptation of *C. jejuni* to cattle could be associated with the presence of genetic elements favoring its survival in the intestine of cattle (Sheppard and Maiden, 2015) and with a significant gene gain and loss (Mourkas et al., 2020). Interestingly, cattle *C. jejuni* showed the strongest association between resistance to aminoglycosides and macrolides (with RR > 25), but only 2 co-resistant isolates (out of 7 co-resistant *C. jejuni* cattle isolates sequenced) were classified into groups 4 and 5.

Among its limitations, the sample size used in the first part of this long study period (2002–2008) was relatively small. Furthermore, AST in the first years (2002–2005) was based on determining IZDs and MICs depending on the antimicrobial considered. Therefore, conclusions based on data from that period must be interpreted carefully. In addition, only 1.5% (168) of the total number of isolates were subjected to the *flaA* gene analysis, and only 51 of them were analyzed by WGS.

Nevertheless, our findings confirm that high resistance levels in *Campylobacter* spp. from food producing animals were consistently observed, and that resistance to macrolides and aminoglycosides was strongly associated across hosts and bacterial species. Further studies based on WGS would be needed in order to determine the genetic determinants behind this resistance and the possible existence of more prevalent lineages.

In this study, *C. coli* isolates, more prevalent in pigs and poultry (especially turkeys), showed significantly higher resistance levels than *C. jejuni* strains. The significant association in the simultaneous presentation of phenotypic resistance to aminoglycosides and macrolides, confirmed in *C. coli* isolates independently from host species of origin, suggests the possible circulation of resistance genes against both antimicrobial classes. Such resistance mechanisms could have been transmitted together, or else, have disseminated via resistant clones in the studied livestock species. The genetic analysis revealed the presence of some isolates more genetically related with resistant phenotypes in poultry and others with susceptible phenotypes in cattle. In order to test these hypotheses it would be necessary to characterize the resistance mechanisms present in isolates from the different species by means of a thorough molecular analysis of their whole genome.

## Data Availability Statement

The original contributions presented in the study are included in the article/Supplementary Material, further inquiries can be directed to the corresponding author.

## Ethics Statement

Ethical review and approval was not required because animals included in this study were sampled in the slaughterhouse during routine processing of livestock and were not subjected to any additional handling of any kind. Samples were collected in the frame of official monitoring programs according to EU and national legislation.

## Author Contributions

VL-C, LD, and JA: conceptualization. VL-C and JA: investigation, writing – original draft preparation, methodology, software, formal analysis, and validation. JA: Funding acquisition and project administration and supervision. JS, CF, TS, IP, MU-R, CB, and MG: data supply. MU-R, CB, and MG: data curation. VL-C and AO: laboratory work. VL-C, MU-R, CB, AO, MG, JS, IP, MM, LD, and JA: writing – review and editing. All authors contributed to the article and approved the submitted version.

## Conflict of Interest

The authors declare that the research was conducted in the absence of any commercial or financial relationships that could be construed as a potential conflict of interest.

## Footnotes

1 EUCAST-European Society of Clinical Microbiology and Infectious Diseases, MIC and zone distributions and ECOFFs http://www.eucast.org/mic_distributions_and_ecoffs/ (accessed 14/02/2020).
