# Supplementary material for: Monitoring of Antimicrobial Resistance to Aminoglycosides and Macrolides in Campylobacter coli and Campylobacter jejuni From Healthy Livestock in Spain (2002–2018)
Source: Front Microbiol. 2021 Jul 2;12:689262. doi: 10.3389/fmicb.2021.689262 (PMC8283307; doi:10.3389/fmicb.2021.689262)
Supplement: Supplementary file 3 [file Data_Sheet_1.docx]

***Supplementary Material***

# Supplementary Data

Supplementary Excel file 1: MIC distributions across host species and *Campylobacter* spp. categories (‘squashtograms’)

Supplementary Excel file 2: Summary of antimicrobial resistance genes retrieved from 51 WGS *Campylobacter* spp. Sequences

# Supplementary Figures and Tables

## Supplementary Figures

**Supplementary Figure 1.** Maps showing distribution and percentage of contribution to national sampling from broilers and turkeys


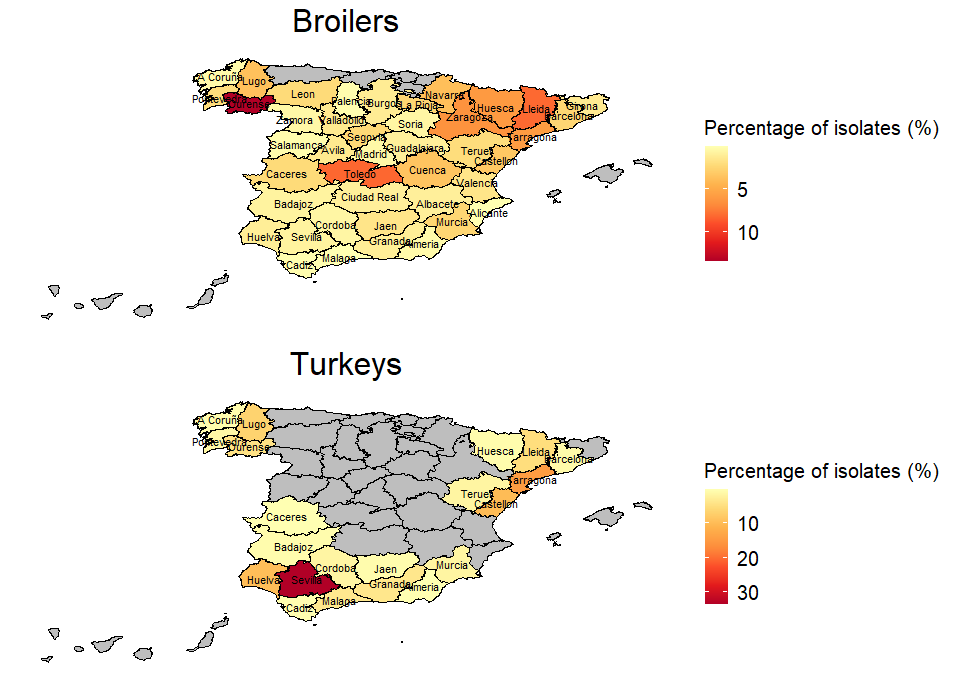


**Supplementary Figure 2.** Maps showing distribution and percentage of contribution to national sampling from pigs and cattle


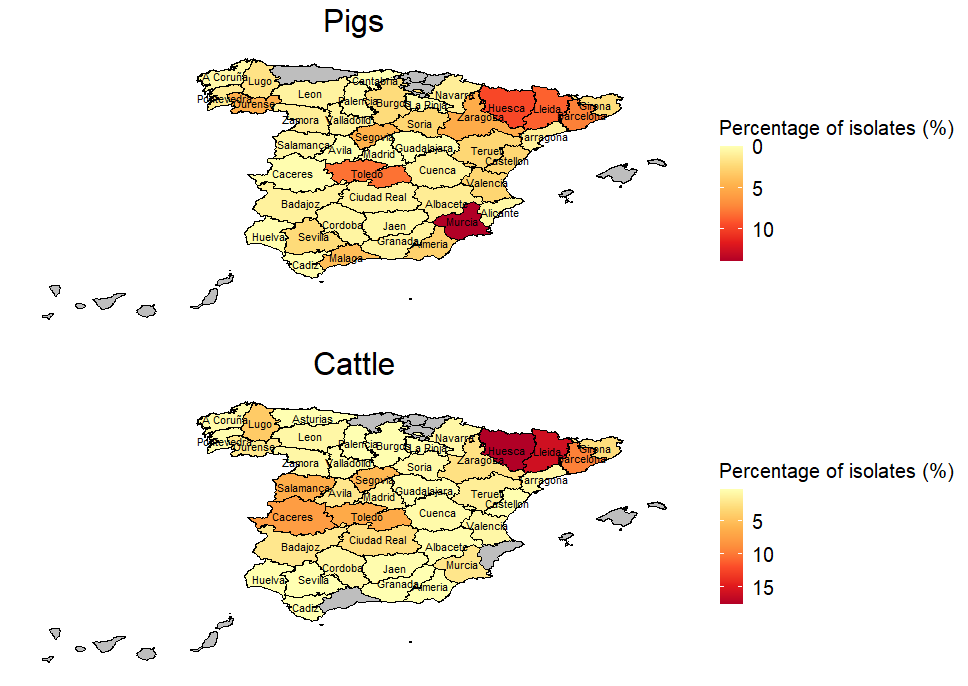


## Supplementary Tables

**Supplementary table 1** – Summary table of *Campylobacter* surveillance in livestock in Spain

| **Year** | **Host Species** | **Sampling Period (# of months/year)** | **# of Abattoirs** | **Average # of Lots (Farms/Abattoir)** | **# of Individual Samples** | **Average # of Farms/**  **Month-Year** | **# of Samples/Lot (Pool)** | **# of Pools (Farms)** | **# of Isolates (All species)** | **# of AST (*C. jejuni* & *C. coli*)** |
| --- | --- | --- | --- | --- | --- | --- | --- | --- | --- | --- |
| 2002 | Pigs | Sep-Dec (4) | 10 | 29 | 580 | 72.5 | 2 | 290 | 97 | 94 |
|  | Broilers | Feb-Jul (6) | 4 | 21.2 | 170 | 14.2 | 3 | 85 | 33 | 29 |
| 2003 | Pigs | Jan-Nov (11) | 10 | 31.9 | 638 | 29 | 2 | 319 | 125 | 113 |
|  | Broilers | Mar-Oct (8) | 9 | 17.8 | 480 | 20 | 3 | 160 | 42 | 32 |
| 2004 | Pigs | Feb-Nov (10) | 8 | 22.9 | 366 | 18.3 | 2 | 183 | 116 | 87 |
|  | Broilers | Feb-Jun (5) | 8 | 19.9 | 477 | 31.8 | 3 | 159 | 52 | 33 |
| 2005 | Pigs | Feb-Dec (11) | 8 | 24.4 | 390 | 17.7 | 2 | 195 | 156 | 140 |
|  | Broilers | Nov-Dec (2) | 6 | 12.7 | 228 | 38 | 3 | 76 | 36 | 31 |
| 2006 | Pigs | Mar-Sep (7) | 8 | 24.5 | 392 | 28 | 2 | 196 | 145 | 100 |
|  | Broilers | May-Sep (5) | 6 | 16.3 | 294 | 19.6 | 3 | 98 | 49 | 46 |
| 2007 | Pigs | Mar-Sep (7) | 8 | 38.7 | 620 | 44.3 | 2 | 310 | 165 | 143 |
|  | Broilers | May-Nov (7) | 7 | 12.7 | 267 | 12.7 | 3 | 89 | 41 | 40 |
|  | Cattle | Jun-Nov (6) | 8 | 20.4 | 326 | 27.2 | 2 | 163 | 75 | 69 |
| 2008 | Pigs | Mar-Dec (10) | 7 | 24.4 | 342 | 17.1 | 2 | 171 | 112 | 93 |
|  | Broilers | Jun-Nov (6) | 7 | 16.1 | 339 | 18.8 | 3 | 113 | 69 | 69 |
|  | Cattle | Jun-Oct (5) | 8 | 21 | 334 | 33.6 | 2 | 168 | 63 | 61 |
| 2009 | Pigs | Apr-Dec (9) | 11 | 25.8 | 568 | 31.5 | 2 | 284 | 192 | 167 |
|  | Broilers | Apr-Dec (9) | 9 | 22 | 594 | 22 | 3 | 198 | 118 | 115 |
|  | Cattle | Apr-Dec (9) | 10 | 25.8 | 516 | 28.7 | 2 | 258 | 107 | 105 |
| 2010 | Pigs | May-Nov (7) | 13 | 16.5 | 428 | 30.6 | 2 | 214 | 135 | 105 |
|  | Broilers | May-Nov (7) | 13 | 15.5 | 2’020 | 28.8 | 10 | 202 | 131 | 127 |
|  | Cattle | May-Nov (7) | 13 | 15.4 | 400 | 28.6 | 2 | 200 | 132 | 130 |
| 2011 | Pigs | May-Dec (8) | 15 | 17.1 | 514 | 32.1 | 2 | 257 | 167 | 129 |
|  | Broilers | May-Dec (8) | 15 | 15.8 | 2’370 | 29.6 | 10 | 237 | 162 | 156 |
|  | Cattle | May-Dec (8) | 15 | 15.9 | 478 | 29.9 | 2 | 239 | 142 | 132 |
| 2012 | Pigs | Jun-Oct (5) | 17 | 9.6 | 326 | 32.6 | 2 | 236 | 163 | 73 |
|  | Broilers | Jun-Oct (5) | 15 | 10.2 | 1’530 | 30.6 | 10 | 239 | 182 | 86 |
|  | Cattle | Jun-Oct (5) | 16 | 9.1 | 292 | 29.2 | 2 | 221 | 151 | 75 |
| 2013 | Pigs | Apr-Oct (7) | 19 | 12.1 | 460 | 32.8 | 2 | 338 | 252 | 108 |
|  | Broilers | Apr-Oct (7) | 15 | 15.3 | 2’300 | 32.8 | 10 | 370 | 284 | 140 |
|  | Cattle | Apr-Oct (7) | 19 | 12.2 | 464 | 33.1 | 2 | 347 | 232 | 115 |
| 2014 | Broilers | Mar-Oct (8) | 15 | 33.3 | 5’000 | 62.5 | 10 | 500 | 268 | 170 |
|  | Turkeys | Mar-Oct (8) | 7 | 71.4 | 5’000 | 62.5 | 10 | 500 | 370 | 170 |
| 2015 | Pigs | May-Nov (7) | 20 | 18.6 | 746 | 53.3 | 2 | 373 | 193 | 170 |
|  | Cattle | May-Nov (7) | 17 | 21.6 | 734 | 52.4 | 2 | 367 | 136 | 120 |
| 2016 | Broilers | Apr-Nov (8) | 17 | 25.1 | 4’270 | 53.4 | 10 | 427 | 257 | 162 |
|  | Turkeys | Apr-Dec (9) | 6 | 81.3 | 4’880 | 54.2 | 10 | 488 | 319 | 170 |
| 2017 | Pigs | Jan-Dec (12) | 19 | 20.2 | 768 | 32 | 2 | 384 | 200 | 170 |
|  | Cattle | Jan-Dec (12) | 24 | 16 | 768 | 32 | 2 | 384 | 228 | 170 |
| 2018 | Broilers | Jan-Dec (12) | 21 | 21.9 | 4’600 | 38.3 | 10 | 460 | 276 | 170 |
|  | Turkeys | Jan-Dec (12) | 5 | 93.4 | 4’670 | 38.9 | 10 | 467 | 401 | 170 |

Source: VAV NETWORK

Samples: Faeces from pigs, broilers, cattle and turkeys (2002-2018)

AST= Antimicrobial susceptibility testing

**Supplementary table 2** – Numbers and proportions of total samples, total isolates and *C. coli* and *C. jejuni* isolates subjected to antimicrobial susceptibility testing (AST) across the four host species

|  | **Broilers** | | | **Turkeys** | | | **Pigs** | | | **Cattle** | | |
| --- | --- | --- | --- | --- | --- | --- | --- | --- | --- | --- | --- | --- |
| Year | Samples | Isolates (%) | AST (%) | Samples | Isolates (%) | AST (%) | Samples | Isolates (%) | AST (%) | Samples | Isolates (%) | AST (%) |
| 2002 | 85 | 33 (38.8) | 29 (34.1) |  | | | 290 | 97 (33.4) | 94 (32.4) |  | | |
| 2003 | 160 | 42 (26.2) | 32 (20.0) |  |  |  | 319 | 125 (39.2) | 113 (35.4) |  |  |  |
| 2004 | 159 | 52 (32.7) | 33 (20.7) |  |  |  | 183 | 116 (63.4) | 87 (47.5) |  |  |  |
| 2005 | 76 | 36 (47.4) | 31 (40.8) |  |  |  | 195 | 156 (80.0) | 140 (71.8) |  |  |  |
| 2006 | 98 | 49 (50.0) | 46 (46.9) |  |  |  | 196 | 145 (74.0) | 100 (51.0) |  |  |  |
| 2007 | 89 | 41 (46.1) | 40 (44.9) |  |  |  | 310 | 165 (53.2) | 143 (46.1) | 163 | 75 (46.0) | 69 (42.3) |
| 2008 | 113 | 69 (61.1) | 69 (61.1) |  |  |  | 171 | 112 (65.5) | 93 (54.4) | 168 | 63 (37.5) | 61 (36.3) |
| 2009 | 198 | 118 (59.6) | 115 (58.1) |  |  |  | 284 | 192 (67.6) | 167 (58.8) | 258 | 107 (41.5) | 105 (40.7) |
| 2010 | 202 | 131 (64.8) | 127 (62.9) |  |  |  | 214 | 135 (63.1) | 105 (49.1) | 200 | 139 (69.5) | 130 (65.0) |
| 2011 | 237 | 162 (68.3) | 156 (65.8) |  |  |  | 257 | 167 (65.0) | 129 (50.2) | 239 | 142 (54.4) | 132 (55.2) |
| 2012 | 239 | 182 (76.1) | 86 (36.0) |  |  |  | 236 | 163 (69.1) | 73 (30.9) | 221 | 151 (68.3) | 75 (33.9) |
| 2013 | 370 | 284 (76.7) | 140 (37.8) |  |  |  | 338 | 252 (74.5) | 108 (31.9) | 347 | 232 (66.8) | 115 (33.1) |
| 2014 | 500 | 268 (53.6) | 170 (34.0) | 500 | 370 (74.0) | 170 (34.0) |  | | |  | | |
| 2015 |  | | |  | | | 373 | 193 (51.7) | 170 (45.6) | 367 | 136 (37.0) | 120 (32.7) |
| 2016 | 427 | 257 (60.2) | 162 (37.9) | 488 | 319 (65.4) | 170 (34.8) |  | | |  | | |
| 2017 |  | | |  | | | 384 | 200 (52.1) | 170 (44.3) | 384 | 228 (59.4) | 170 (44.3) |
| 2018 | 460 | 276 (60.0) | 170 (36.9) | 467 | 401 (85.9) | 170 (36.4) |  | | |  | | |
| **TOTAL** | **3,413** | **2,000 (58.6)** | **1,406 (41.2)** | **1,455** | **1,090 (74.9)** | **510 (35.0)** | **3,750** | **2,218 (59.1)** | **1,692 (45.1)** | **2,347** | **1,273 (54.2)** | **977 (41.6)** |

**Supplementary table 3** – Antimicrobial phenotypic non-susceptibility (resistance) profiles across animal host species in *C. coli* and *C. jejuni*

| **Number** | **Antimicrobials** | ***C. coli* Broilers** | ***C. coli* Turkeys** | ***C. coli***  **Pigs** | ***C. coli***  **Cattle** | ***C. jejuni* Broilers** | ***C. jejuni* Turkeys** | ***C. jejuni* Cattle** | **Number of isolates** |
| --- | --- | --- | --- | --- | --- | --- | --- | --- | --- |
| 0 | Pan-susceptible | 7 (1.10) | 2 (0.72) | 1 (0.06) | 5 (3.35) | 46 (5.96) | 14 (6.06) | 129 (15.58) | 204 (4.45) |
| 1 | CIP\|TET\|NAL\|ERY\|STR | 8 (1.26) | 0 (0) | 7 (0.41) | 5 (3.35) | 32 (4.14) | 9 (3.90) | 157 (18.96) | 218 (4.75) |
| 2 | CIP/NAL | 12 (1.89) | 4 (1.43) | 0 (0) | 1 (0.67) | 73 (9.46) | 24 (10.39) | 66 (7.97) | 180 (3.93) |
|  | (GEN\|CIP)/TET | 28 (4.42) | 6 (2.15) | 2 (0.12) | 0 (0) | 70 (9.07) | 7 (3.03) | 14 (1.69) | 127 (2.77) |
|  | TET/STR | 14 (2.21) | 1 (0.36) | 47 (2.78) | 9 (6.04) | 2 (0.26) | 1 (0.43) | 7 (0.84) | 81 (1.77) |
|  | OTHERS | 2 (0.31) | 0 (0) | 7 (0.41) | 0 (0) | 2 (0.26) | 2 (0.86) | 5 (0.60) | 18 (0.39) |
| 3 | CIP/TET/NAL | **160 (25.24)** | **76 (27.24)** | 40 (2.36) | 14 (9.39) | **474 (61.40)** | **159 (68.83)** | **374 (45.17)** | 1,297 (28.29) |
|  | TET/ERY/STR | 1 (0.16) | 1 (0.36) | 56 (3.31) | 0 (0) | 0 (0) | 0 (0) | 0 (0) | 58 (1.26) |
|  | CIP/TET/(ERY\|STR) | 39 (6.15) | 3 (1.07) | 2 (0.12) | 1 (0.67) | 3 (0.39) | 0 (0) | 0 (0) | 48 (1.05) |
|  | OTHERS | 10 (1.58) | 1 (0.36) | 13 (0.77) | 1 (0.67) | 2 (0.26) | 0 (0) | 7 (0.84) | 34 (0.74) |
| 4 | CIP/TET/NAL/STR | **119 (18.77)** | **77 (27.60)** | 371 (21.93) | **75 (50.33)** | 47 (6.09) | 11 (4.76) | 52 (6.28) | 752 (16.40) |
|  | CIP/TET/NAL/ERY | 48 (7.57) | 30 (10.75) | 84 (4.96) | 1 (0.67) | 10 (1.29) | 2 (0.86) | 1 (0.12) | 176 (3.84) |
|  | OTHERS | 21 (3.31) | 2 (0.72) | 18 (1.06) | 0 (0) | 3 (0.39) | 0 (0) | 2 (0.24) | 46 (1.00) |
| 5 | CIP/TET/NAL/ERY/STR | 84 (13.25) | 56 (20.07) | **687 (40.60)** | 19 (12.75) | 4 (0.52) | 2 (0.86) | 5 (0.60) | 857 (18.69) |
|  | GEN/CIP/TET/NAL/STR | 28 (4.42) | 9 (3.22) | 77 (4.55) | 9 (0.64) | 1 (0.13) | 0 (0) | 5 (0.60) | 129 (2.81) |
|  | OTHERS | 7 (1.10) | 1 (0.36) | 14 (0.83) | 0 (0) | 1 (0.13) | 0 (0) | 0 (0) | 23 (0.50) |
| 6 | Pan-resistant | 46 (7.25) | 10 (3.58) | 266 (15.72) | 9 (0.64) | 2 (0.26) | 0 (0) | 4 (0.48) | 337 (7.35) |
|  | TOTAL | 634 (100) | 279 (100) | 1,692 (100) | 149 (100) | 772 (100) | 231 (100) | 828 (100) | 4,585 (100) |

In bold = proportions of resistotypes/isolates larger than 25% within each category

GEN=Gentamicin; CIP=Ciprofloxacin; TET=Tetracycline; NAL=Nalidixic acid; ERY=Erythromycin; STR=Streptomycin
